# Anti-Oxidant and Anti-Inflammatory Effects of Lipopolysaccharide from *Rhodobacter sphaeroides* against Ethanol-Induced Liver and Kidney Toxicity in Experimental Rats

**DOI:** 10.3390/molecules26247437

**Published:** 2021-12-08

**Authors:** Eman T. Mehanna, Al-Shimaa A. Ali, Fatma El-Shaarawy, Noha M. Mesbah, Dina M. Abo-Elmatty, Nora M. Aborehab

**Affiliations:** 1 Department of Biochemistry, Faculty of Pharmacy, Suez Canal University, Ismailia 41522, Egypt; dr.shimoo18@yahoo.com (A.-S.A.A.); noha_mesbah@pharm.suez.edu.eg (N.M.M.); dinawahadan@yahoo.com (D.M.A.-E.); 2 Department of Biochemistry, Faculty of Pharmacy, Sinai University, El Arish 45518, Egypt; fatma.mohamed@su.edu.eg; 3 Department of Biochemistry, Faculty of Pharmacy, October University for Modern Science and Arts (MSA), 6th October City 12451, Egypt; naborehab@msa.edu.eg

**Keywords:** alcoholic liver disease, hepcidin, kidney injury molecule-1, lipopolysaccharide from *Rhodobacter sphaeroides*, nuclear factor kappa B, toll-like receptor 4

## Abstract

This study aimed to investigate the protective effects of lipopolysaccharide from *Rhodobacter sphaeroides* (LPS-RS) against ethanol-induced hepatotoxicity and nephrotoxicity in experimental rats. The study involved an intact control group, LPS-RS group, two groups were given ethanol (3 and 5 g/kg/day) for 28 days, and two other groups (LPS-RS + 3 g/kg ethanol) and (LPS-RS + 5 g/kg ethanol) received a daily dose of LPS-RS (800 μg/kg) before ethanol. Ethanol significantly increased the expression of nuclear factor kappa B (NF-κB) and levels of malondialdehyde (MDA), tumor necrosis factor-alpha (TNF-α) and interleukin-6 (IL-6) in the liver tissue and decreased anti-oxidant enzymes. Hepcidin expression was downregulated in the liver, with increased serum levels of ferritin and iron. Prior-administration of LPS-RS alleviated the increase in oxidative stress and inflammatory markers, and preserved iron homeostasis markers. In the kidney, administration of ethanol caused significant increase in the expression of NF-κB and the levels of TNF-α and kidney injury markers; whereas LPS-RS + ethanol groups had significantly lower levels of those parameters. In conclusion; this study reports anti-oxidant, anti-inflammatory and iron homeostasis regulatory effects of the toll-like receptor 4 (TLR4) antagonist LPS-RS against ethanol induced toxicity in both the liver and the kidney of experimental rats.

## 1. Introduction

Alcoholic liver disease (ALD) is a worldwide health problem which may result in the development of hepatitis, fatty liver steatosis and cirrhosis [1]. Alcohol is known to exert a harmful effect on a variety of human tissues. In particular, the liver is the major site of alcohol-induced damage because it is the direct recipient of the blood that contains elevated levels of alcohol, and it is the main organ responsible for the metabolism of alcohol [2]. The damage caused by ethanol is mainly attributed to its metabolic process that results in generation of acetaldehyde and reactive oxygen species (ROS) such as hydrogen peroxide, free hydroxyl radical, and superoxide. These metabolites cause depletion of reduced glutathione (GSH), peroxidation of cellular membranes, oxidation of macro-molecules, and eventually lead to progressive injury of hepatocytes [3,4,5]. Additionally, ethanol and its metabolic products enhance the production of inflammatory cytokines such as interleukin-6 (IL-6) and tumor necrosis factor-alpha (TNF-α) [6]. The enhanced production of those inflammation factors; stimulated partially by oxidative stress; results in cytokine imbalance and immune disorders, leading to further hepatic damage. Thus, agents with anti-inflammatory and anti-oxidative properties might be potential candidates for protection against alcohol-induced liver disease [7,8].

The metabolic functions of the alcoholic liver are seriously affected. Disorders in iron metabolism are characteristic of ALD. Abnormal levels of iron, transferrin, and ferritin were detected in ALD [9]. Hepcidin is a principle hepatic regulator of the metabolism of iron. The expression of hepcidin in the liver is downregulated by both acute and chronic exposure to alcohol [10,11]. Ethanol consumption increases absorption of iron, presumably by downregulation of hepcidin expression, leading to increased ferritin levels [12,13]. The increased levels of iron and ferritin may play a role in the progression of ALD to cirrhosis and eventually hepatocellular carcinoma [14].

The oxidative stress and the inflammatory state caused by excessive intake of alcohol may cause damage to other organs as well. The kidney is one organ whose structure and functions are markedly affected by ethanol [15]. Chronic administration of ethanol affects renal function and inhibits renal tubular reabsorption. Alcohol consumption was reported to cause abnormal thickening of the basement membrane of the glomeruli due to enhanced cell proliferation and inflammation in the cells of the kidney tubules. These deleterious effects result in impairment of the kidney’s ability to regulate the body’s fluid volume and electrolyte balance [16,17].

Lipopolysaccharide from *Rhodobacter sphaeroides* (LPS-RS) is a penta-acylated lipid A that inhibits the toll-like receptor 4 (TLR4) pathway by two mechanisms; the first is based on the direct competition for binding on MD-2 between hexa-acylated lipid A and underacylated lipid A, and the other relies on the inhibition of hexa-acylated endotox-in:MD-2 complexes and TLR4 functions by penta-acylated lipid A:MD-2 complexes. LPS-RS regulates c-Jun N-terminal kinase (JNK)/p38 MAPKs and p65-NF-κB signaling pathways and inhibits TLR4 mediated inflammatory markers such as TNF-α, IL-1β, and IL-6 [18].

The current work aimed to assess the potential protective effects of LPS-RS administration against the liver and kidney damage caused by chronic consumption of moderate and high doses of ethanol in experimental rats. The anti-oxidative and anti-inflammatory activities of LPS-RS and its effect on hepatic iron metabolism in a rat model of ALD were investigated.

## 2. Results and Discussion

### 2.1. Effect of LPS-RS on Serum Levels of Liver Enzymes and Kidney Function Markers

Table 1 shows that administration of LPS-RS alone had no adverse effects on both the liver and the kidney as indicated by the assessed biochemical parameters. Administration of either 3 g/kg or 5 g/kg of ethanol led to a significant increase in the serum levels of alanine aminotransferase (ALT), aspartate aminotransferase (AST) and alkaline phosphatase (ALP) compared to the intact control group. The increase in the levels of those markers in the group receiving 5 g/kg ethanol was significantly higher than the group receiving 3 g/kg ethanol. LPS-RS had a protective effect, where the levels of ALT, AST and ALP were significantly decreased in both LPS-RS + ethanol (3 g/kg) and LPS-RS + ethanol (5 g/kg) groups compared to the corresponding unprotected groups (i.e., ethanol (3 g/kg) and ethanol (5 g/kg), respectively) (Table 1).

Similarly, serum creatinine and blood urea nitrogen (BUN) levels were significantly raised in both ethanol (3 g/kg and 5 g/kg) groups compared to the intact control group. The administration of LPS-RS in both groups led to a significant decrease in the level of serum creatinine but not the level of BUN (Table 1).

### 2.2. Effect of LPS-RS on Oxidative Stress and Inflammatory Markers in the Liver Tissue

**Figure 1:**
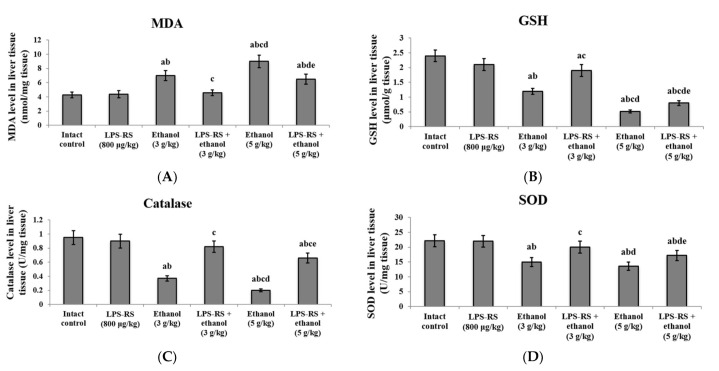
The anti-oxidant effects of LPS-RS (800 μg/kg) in the liver tissue of the experimental rats. Levels of (**A**) MDA, (**B**) GSH, (**C**) catalase, and (**D**) SOD. MDA, malondialdehyde; GSH, reduced glutathione; SOD, superoxide dismutase. Data are expressed as mean ± SD and analyzed using one-way ANOVA followed by Bonferroni’s post-hoc test (*n* = 8–10). Differences were considered significantly different at *p* < 0.05. ^a^ vs. intact control; ^b^ vs. LPS-RS; ^c^ vs. ethanol (3 g/kg); ^d^ vs. LPS-RS + ethanol (3 g/kg); ^e^ vs. ethanol (5 g/kg).

Daily administration of 800 μg/kg LPS-RS only did not cause a significant difference in any of the markers of oxidative stress or inflammatory state in the liver tissue in comparison with the intact control group (Figure 1). MDA; indicative of lipid peroxidation; was significantly increased in both ethanol (3 g/kg and 5 g/kg) groups compared to the intact control group. The increase was significantly higher in the 5 g/kg ethanol group compared to the 3 g/kg ethanol group. Administration of LPS-RS prior to ethanol in both groups caused a significant decrease in the MDA levels compared to the corresponding unprotected groups (Figure 1A).

Levels of GSH and the anti-oxidant enzymes; catalase and SOD; in the liver tissue were significantly decreased by administration of both doses of ethanol compared to the intact control group. The levels of GSH and catalase in the 5 g/kg ethanol group were significantly lower compared to the ethanol (3 g/kg) group. Administration of 800 μg/kg of LPS-RS before ethanol (3 or 5 g/kg) led to a significant increase of GSH, catalase and SOD levels compared to the corresponding unprotected groups (Figure 1B–D).

**Figure 2:**
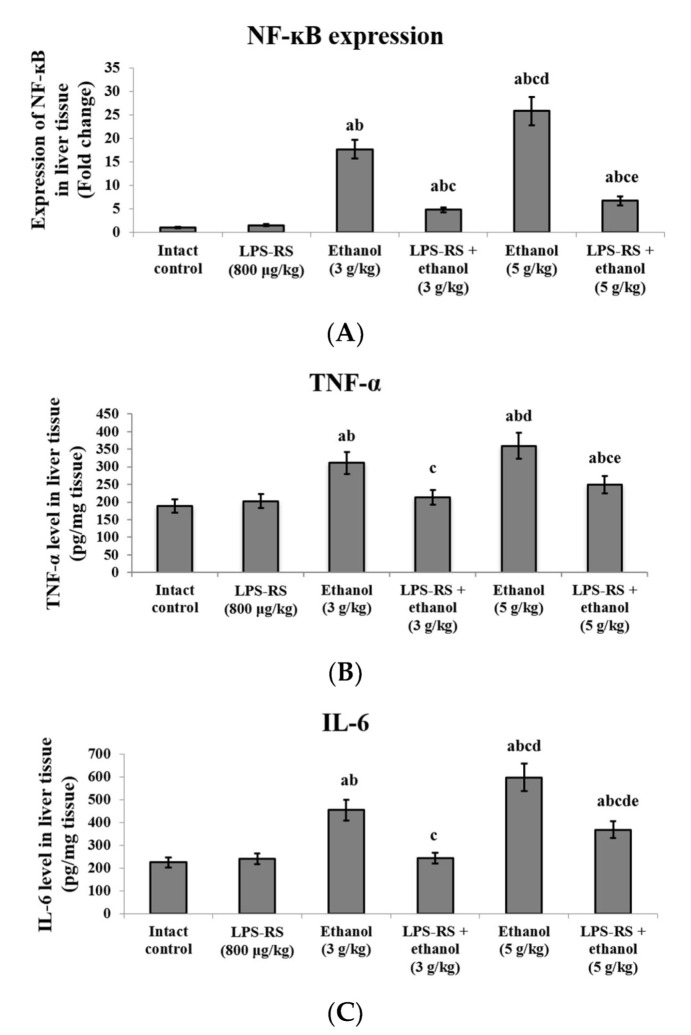
The effect of LPS-RS (800 μg/kg) on inflammatory markers in the liver tissue of the experimental rats. (**A**) the expression of NF-kB, (**B**) levels of TNF-α and (**C**) levels of IL-6. NF-κB, nuclear factor kappa B; TNF-α, tumor necrosis factor-alpha; IL-6, interleukin-6. Data are expressed as mean ± SD and analyzed using one-way ANOVA followed by Bonferroni’s post-hoc test (*n* = 8–10). Differences were considered significantly different at *p* < 0.05. ^a^ vs. intact control; ^b^ vs. LPS-RS; ^c^ vs. ethanol (3 g/kg); ^d^ vs. LPS-RS + ethanol (3 g/kg); ^e^ vs. ethanol (5 g/kg).

Expression of NF-κB and levels of inflammatory markers TNF-α and IL-6 were assessed in the liver tissue of all experimental groups (Figure 2). Ethanol administration caused a 17.7-fold increase in expression of NF-κB in the liver in the 3 g/kg ethanol group, and a 25.8-fold increase in expression in the 5 g/kg ethanol group compared to the intact control group. LPS-RS administration prior to ethanol downregulated the expression of NF-κB by 3.7 fold in LPS-RS + ethanol (3 g/kg) group and by 3.9 fold in LPS-RS + ethanol (5 g/kg) group relative to the corresponding unprotected groups (Figure 2A). Levels of both TNF-α and IL-6 were increased significantly by administration of ethanol (3 g/kg or 5 g/kg) compared to the intact control group. IL-6 levels were significantly higher in the group that received 5 g/kg ethanol compared to the 3 g/kg ethanol group. Administration of LPS-RS in both groups caused a significant decrease of the levels of both TNF-α and IL-6 compared to the groups that received ethanol only (Figure 2B,C).

Liver is the major organ that metabolizes alcohol. The metabolism of alcohol in the liver leads to production of metabolites and byproducts that impair lipid metabolism and intensify inflammatory reactions in the liver [19]. The metabolism of alcohol by cytochrome P450 2E1 in the liver leads to excessive production of reactive oxygen radicals and induction of endoplasmic reticulum stress [20,21], which in turn deteriorates the lysosomal function and autophagy and results ultimately in mitochondrial injury and hepatocellular death [22]. Oxidative stress triggers adaptive immune responses in ALD [7]. Alcohol consumption activates the cluster of differentiation 14 (CD14)/TLR4 pathway which can induce cellular injury through activation of macrophages and generation of inflammatory mediators such as IL1β, IL-6 and TNF-α [23,24]. Oxidative stress pathways are suggested to play an essential role in regulating the production of the cytokines and chemokines that are activated by the TLR4/NF-κB pathway [22].

TLR4 is a major pathway that induces inflammation TLR4 is expressed mainly in macrophages and its activation initiates intracellular signaling through phosphorylation of mitogen-activated protein kinases (MAPKs) and NF-κB, inducing the expression of inflammatory cytokines such as IL-6 and TNF-α [25,26]. These inflammatory factors accelerate oxidative stress and decrease the antioxidant capacity of cells through reducing SOD activity and increasing MDA production [27,28]. Moreover, the decreased antioxidant enzyme levels may be attributed to their over-consumption in alleviation of the oxidative stress that is caused by the accelerated inflammation [29].

LPS-RS is a potent TLR4 antagonist that attenuates TLR-4 mediated inflammation [18]. The binding of LPS-RS to the complex of TLR4/myeloid differentiation factor-2 (MD-2), either in vitro or in vivo, inhibits the nuclear factor kappa B (NF-κB) mediated generation of inflammatory factors such as TNF-α and IL-6 [30].

In agreement with the results of the current study, LPS-RS was reported to attenuate inflammation and increase GSH levels and SOD activity in LPS-induced acute lung injury [31]. LPS-RS also modulated the release of cytokines and exerted analgesic effect in a rat neuropathic model through blocking TLR4 expression [32]. Additionally, a recent study reported that TLR4 inactivation by LPS-RS attenuated chronic inflammation in middle ear cholesteatoma stem cells, resulting in a significant decrease of TNF-α, IL-6, and IL-1ß expression [33].

### 2.3. Effect of LPS-RS on the Expression of Hepcidin in the Liver Tissue and Serum Levels of Iron and Ferritin

**Figure 3:**
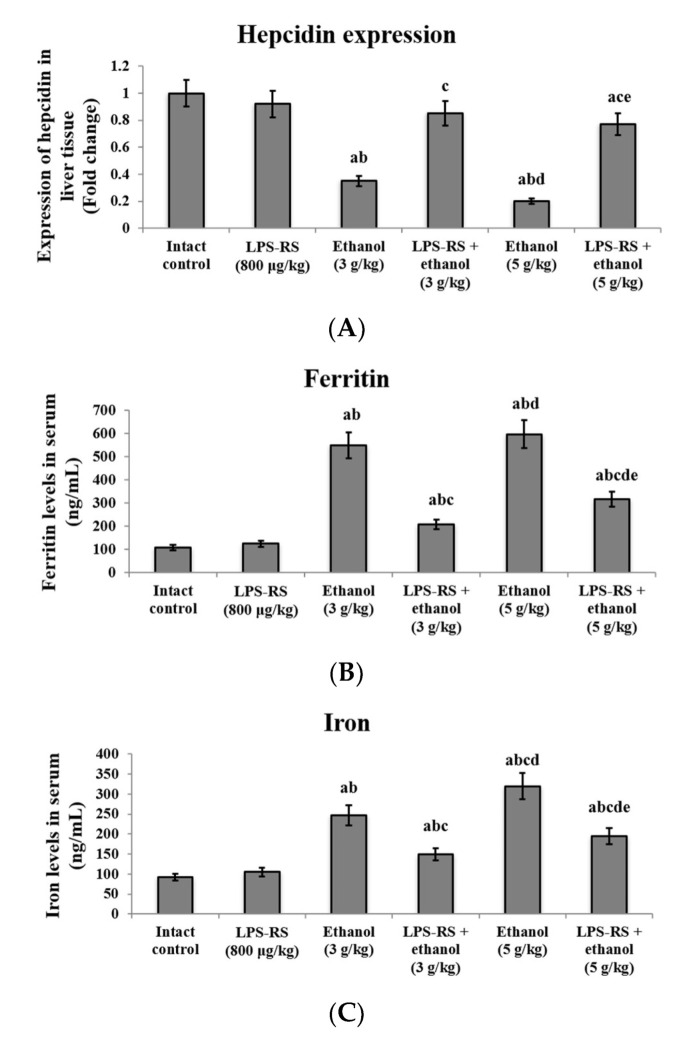
The effect of LPS-RS (800 μg/kg) on the iron homeostasis markers in the experimental rats. (**A**) the expression of hepcidin in liver tissue, serum levels of (**B**) ferritin and (**C**) iron. Data are expressed as mean ± SD and analyzed using one-way ANOVA followed by Bonferroni’s post-hoc test (*n* = 8–10). Differences were considered significantly different at *p* < 0.05. ^a^ vs. intact control; ^b^ vs. LPS-RS; ^c^ vs. ethanol (3 g/kg); ^d^ vs. LPS-RS + ethanol (3 g/kg); ^e^ vs. ethanol (5 g/kg).

Expression of hepcidin was quantified in the hepatic tissue by real-time PCR. Administration of ethanol (3 g/kg and 5 g/kg) decreased the expression of hepcidin by 2.9 and 5 fold, respectively, in comparison to the intact control group. LPS-RS administration in both groups significantly upregulated its expression relative to the corresponding unprotected group. Notably, the upregulation of hepcidin was more effective in the LPS-RS + ethanol (3 g/kg), where the expression of hepcidin showed no significant difference compared to the normal control group (Figure 3A).

Serum levels of iron and ferritin were also determined. A significant increase in their levels was recorded in both the ethanol administered groups (3 and 5 g/kg) compared to the intact control group, with the levels of iron in the 5 g/kg ethanol group significantly higher than those in the 3 g/kg ethanol group. Serum levels of iron and ferritin were reduced significantly in the two groups that received LPS-RS prior to ethanol (Figure 3B,C) (Figure 3).

Hepcidin is a liver-synthesized hormone that acts to regulate iron. Inflammation, erythropoiesis, and iron modulate the expression of hepcidin [34]. The oxidative stress created by alcohol consumption inhibits the promoter activity and transcription of hepcidin in the hepatocytes, causing accumulation of iron in the liver [35]. Alcohol inhibits hepcidin expression through suppression of CCAAT-enhancer-binding protein in hepatocytes and counteracting its induction by iron [36]. Alcohol was also postulated to suppress hepcidin expression through stimulating TLR4 signaling and NF-кB in the presence of inflammation in the liver. Interestingly, alcohol could not suppress hepcidin expression in mice with defective TLR4 receptor [37,38], which agrees with the findings of the current study.

Another major player in iron storage and transport is ferritin; which is produced in the liver by hepatocytes, Kupffer cells, and macrophages [39]. Ferritin is a marker of iron bioavailability and its synthesis is induced by high iron levels. Ferritin also serves as an acute phase reactant as the proinflammatory cytokines IL-1 and TNF-α increase its hepatic synthesis [40,41]. Excessive hepatic stores of iron, caused by excessive alcohol consumption, may cause oxidative stress-induced damage and promote hepatic fibrosis [42,43]. Excessive ferritin binds to specific receptors on hepatic stellate cells leading to increased hepatic collagen deposition [44], and activation of NF-κB, that promotes synthesis of inflammatory mediators [45]. In the current study, LPS-RS administration before ethanol provided protection against the ethanol-induced abnormalities in the levels of those iron homeostasis parameters.

### 2.4. Effect of LPS-RS on Markers of Inflammation and Kidney Injury in the Renal Tissue

**Figure 4:**
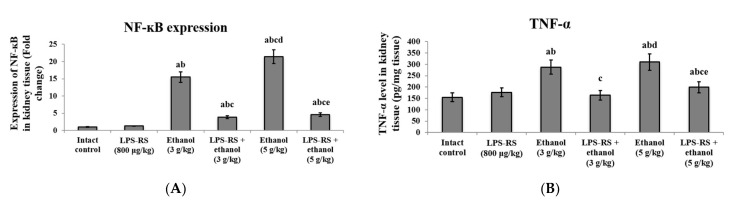
The effect of LPS-RS (800 μg/kg) on inflammatory markers in the kidney tissue of the experimental rats. (**A**) the expression of NF-kB, and (**B**) levels of TNF-α. NF-κB, nuclear factor kappa B; TNF-α, tumor necrosis factor-alpha. Data are expressed as mean ± SD and analyzed using one-way ANOVA followed by Bonferroni’s post-hoc test (*n* = 8–10). Differences were considered significantly different at *p* < 0.05. ^a^ vs. intact control; ^b^ vs. LPS-RS; ^c^ vs. ethanol (3 g/kg); ^d^ vs. LPS-RS + ethanol (3 g/kg); ^e^ vs. ethanol (5 g/kg).

Ethanol administration caused a significant increase of the inflammatory markers in the kidney tissue (Figure 4) Expression of NF-κB was upregulated by 15.5 fold and 21.4 fold in the 3 g/kg and 5 g/kg ethanol groups, respectively, compared to the intact control group. LPS-RS administration prior to 3 g/kg and 5 g/kg ethanol downregulated the expression of NF-κB by 4 fold and by 4.7 fold, respectively (Figure 4A). Level of TNF-α in kidney tissue was also significantly increased in both groups that received ethanol only compared to the intact control group, and was significantly decreased in the groups that received a protective dose of LPS-RS compared to the unprotected groups (Figure 4B).

Chronic administration of ethanol promotes hyperacetylation of mitochondrial proteins and increases oxidative stress in the kidneys, resulting in metabolic dysregulation, and impaired renal function [46]. The TLR4/NF-kB signaling pathway is a significant mediator of inflammation and fibrosis in renal injury [47]. TLR4 is expressed in several renal cells, including endothelial cells, tubular epithelial cell, podocytes, and mesangial cells. The transcription of various chemokines and pro-inflammatory cytokines is regulated by TLR4 signaling, leading to renal inflammation [48,49]. The levels of pro-inflammatory mediators such as TNF-α in renal parenchymal cells are increased through activation of the p38 MAPK pathways by TLR4 signaling [50]. Targeting TLR4 and its downstream mediators may represent an effective approach to alleviate renal inflammation and subsequent kidney injury [48]. Matching with our results, TLR4/NF-κB pathway inhibition and regulation of oxidative stress were previously reported to attenuate other experimental models of nephrotoxicity [51,52].

**Figure 5:**
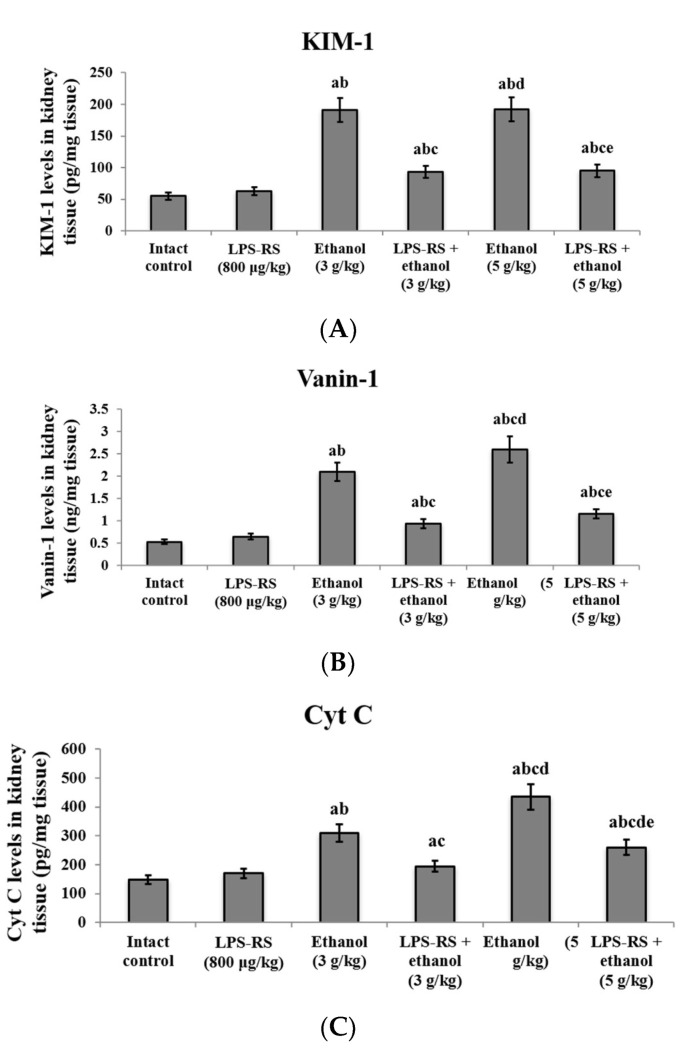
The effect of LPS-RS (800 μg/kg) on the levels of biochemical markers of kidney injury. (**A**) KIM-1, (**B**) Vanin-1 and (**C**) Cyt C in kidney tissue of the experimental rats. KIM-1, kidney injury molecule-1; Vanin-1, vascular non-inflammatory molecule-1; Cyt C, cytochrome C. Data are expressed as mean ± SD and analyzed using one-way ANOVA followed by Bonferroni’s post-hoc test (*n* = 8–10). Differences were considered significantly different at *p* < 0.05. ^a^ vs. intact control; ^b^ vs. LPS-RS; ^c^ vs. ethanol (3 g/kg); ^d^ vs. LPS-RS + ethanol (3 g/kg); ^e^ vs. ethanol (5 g/kg).

Additionally, Markers of tissue injury were determined in the kidney tissue in the current work. Kidney injury molecule-1 (KIM-1), vascular non-inflammatory molecule-1 (vanin-1) and cytochrome C (Cyt C) were all increased significantly by administration of ethanol (3 and 5 g/kg). Levels of vanin-1 and Cyt C in the 5 g/kg ethanol group were significantly higher than their levels in the 3 g/kg ethanol group. LPS-RS administration in both groups caused a significant decrease in the levels of the three markers compared to the groups that did not receive LPS-RS (Figure 5).

KIM-1 is considered as an early biomarker in cases of acute kidney injury [53], and is a sensitive biomarker for chronic kidney disease [54]. KIM-1 promotes production of proinflammatory chemokines [55], and activates macrophage activation through the MAPK signaling pathway in kidney. This is consistent with the increased circulating levels of the macrophage inflammatory markers, such as IL-6 and TNF-α [56].

Vanin-1 is a protein that is highly expressed in various organs, including the kidney, intestine, and liver [57]. Many studies have recently elucidated its association with inflammation and oxidative stress both in physiological and pathophysiological conditions [58,59]. In agreement with our results, increased vanin-1 levels and expression were reported in several models of induced renal toxicity in experimental animals, suggesting a potential role as a biomarker of renal injury [59]. Increased levels of vanin-1 along with upregulated p38 MAPK and NF-kB were detected in mice with severe renal inflammation and fibrosis [60].

Cyt C is a mitochondrial protein that is released from damaged cells into the extracellular space. Cyt C indicates death of cells and its release indicates mitochondrial damage that is associated with cellular apoptosis and/or necrosis [61]. Cyt C has been suggested as a non-invasive biomarker of xenobiotics-induced renal injury [62]. Extracellular Cyt C was hypothesized to interact with the TLR4 in human astrocytes inducing release of inflammatory cytokines [63]. In a model of diabetic mice, blockage of TLR4 was reported to reverse the increased renal expression of Cyt C [64], which supports the results of the current work.

### 2.5. Histopathological Examination of the Liver and Kidney Tissues

**Figure 6:**
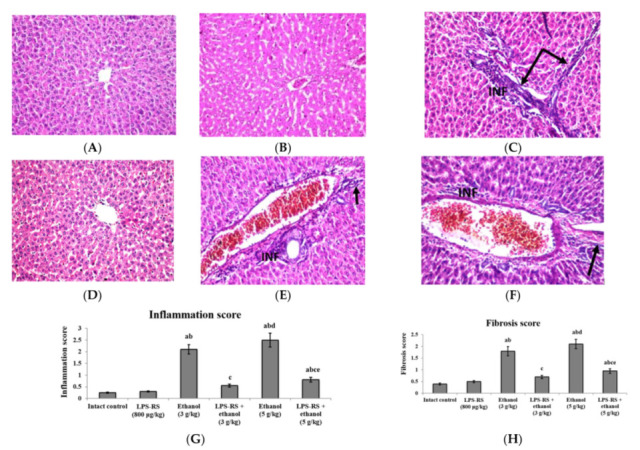
Histopathological sections of rats’ liver stained with hematoxylin and eosin (H&E) (40×). (**A**) intact control group and (**B**) LPS-RS group; showing intact superficial layer, normal population and orientation of hepatocytes, (**C**) rats receiving ethanol (3 g/kg); dilation was observed in the central vein associated with inflammatory cells infiltration (INF) and fibrosis (black arrows) in the portal area, (**D**) LPS-RS + ethanol (3 g/kg) group; showing almost normal histopathological structure, (**E**) rats receiving ethanol (5 g/kg); the portal area showed congestion in the portal vein with fibrosis (black arrow) and few inflammatory cells infiltration (INF), diffuse Kupffer cells proliferation as well as diffuse inflammatory cells infiltration (INF) were detected in between the hepatocytes, (**F**) LPS-RS + ethanol (5 g/kg) group; dilation and congestion were detected in the central veins with few inflammatory cells infiltration (INF) in the portal area, (**G**) Inflammation score, and (**H**) fibrosis score. Scores are expressed as mean ± SD and analyzed using one-way ANOVA followed by Bonferroni’s post-hoc test (*n* = 8–10). Differences were considered significantly different at *p* < 0.05. ^a^ vs. intact control; ^b^ vs. LPS-RS; ^c^ vs. ethanol (3 g/kg); ^d^ vs. LPS-RS + ethanol (3 g/kg); ^e^ vs. ethanol (5 g/kg).

**Figure 7:**
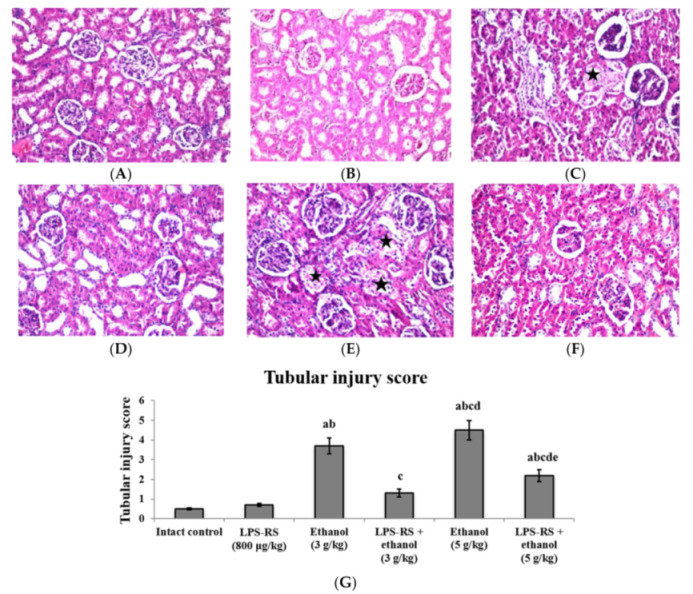
Histopathological sections of rats’ kidney stained with hematoxylin and eosin (H&E) (40×). (**A**) intact control group and (**B**) LPS-RS group; showing intact superficial layer, normal population and orientation of nephrocytes, (**C**) rats receiving ethanol (3 g/kg); showing coagulative necrosis (black star) in the lining epithelium of some individual tubules at the cortex, (**D**) LPS-RS + ethanol (3 g/kg) group; showing normal histopathological structure in kidney tissue, (**E**) rats receiving ethanol (5 g/kg); showing coagulative necrosis (black stars) in the lining epithelial cells of some individual tubules, (**F**) LPS-RS + ethanol (5 g/kg) group; showing almost normal histopathological structure, (**G**) Tubular injury score. The score is represented as mean ± SD and analyzed using one-way ANOVA followed by Bonferroni’s post-hoc test (*n* = 8–10). Differences were considered significantly different at *p* < 0.05. ^a^ vs. intact control; ^b^ vs. LPS-RS; ^c^ vs. ethanol (3 g/kg); ^d^ vs. LPS-RS + ethanol (3 g/kg); ^e^ vs. ethanol (5 g/kg).

Histopathological examination of both the liver and the kidney tissues showed normal structure in both the intact control group and the group that received LPS-RS only (Figure 6 and Figure 7). The liver tissue in the group that received 3 g/kg ethanol showed dilation in the central vein combined with fibrosis and inflammatory cells infiltration. The group that received 5 g/kg ethanol showed congestion in the portal vein with fibrosis and inflammatory cells infiltration. Diffuse Kupffer cells proliferation as well as diffuse inflammatory cells infiltration were observed between the hepatocytes. The pathological changes were attenuated by administration of LPS-RS, especially in the LPS-RS + ethanol (3 g/kg) group that showed almost normal structure (Figure 6). Hepatic fibrosis and inflammation in both LPS treated groups were significantly decreased. Inflammation and fibrosis score of the group that received LPS-RS + ethanol (3 g/kg) was not significantly different compared to the intact control group (Figure 6G,H).

Similarly, histopathological examination of the kidney tissue demonstrated coagulative necrosis in the lining epithelium of some individual tubules at the cortex in both groups that received ethanol only. These effects were diminished in both groups that received LPS-RS prior to ethanol (Figure 7). Tubular injury score in both groups that received LPS was significantly lower than the unprotected groups. Decreased tubular injury was mostly pronounced in the LPS-RS + ethanol (3 g/kg) group (Figure 7G).

## 3. Materials and Methods

### 3.1. Experimental Animals

The current study was conducted on 60 male albino rats (180–200 g weight). The experimental rats were obtained from the Egyptian Organization for Biological Products and Vaccines (Cairo, Egypt), and kept in the animal house of Faculty of Pharmacy, Cairo University under experimentally optimized conditions (i.e., 25 ± 4 °C temperature and normal light/dark cycle) with free access to food and water ad libitum. The animals were kept for a week before the experiment to acclimatize. The study protocol has been approved by the research ethics committee at Faculty of Pharmacy, Suez Canal University, Ismailia, Egypt (Ethics code: code 201912RA3) in agreement with the Guidelines of Canadian Council on Animal Care.

### 3.2. Study Design

The experimental rats were divided randomly into six groups (10 rats each) as follows:

Group I: The intact control group; received 2.5 mL/kg of the phosphate-buffered saline (PBS) vehicle intraperitoneally (i.p.).

Group II: Rats received a daily 800 μg/kg i.p. dose of LPS-RS.

Group III: Rats received ethanol (70% *w*/*v*) daily at a dose of 3 g/kg via intra-gastric gavage [65].

Group IV: LPS-RS + ethanol (3 g/kg); received a daily 800 μg/kg i.p. dose of LPS-RS 30 min prior to intra-gastric administration of 3 g/kg ethanol [66].

Group V: Rats received ethanol (70% *w*/*v*) daily at a dose of 5 g/kg through intra-gastric gavage [67].

Group VI: LPS-RS + ethanol (5 g/kg); similar to group III; 800 μg/kg LPS-RS were administered i.p. before the daily intra-gastric dose of ethanol (5 g/kg) for 28 days.

Ethanol and LPS-RS were acquired from Sigma-Aldrich (Darmstadt, Germany). LPS-RS was dissolved in PBS vehicle. Ethanol and LPS-RS were administered daily for 28 consecutive days. The volume of injected ethanol was calculated based on the density of 70% ethanol at 20 °C (≈0.880 g/mL) as determined by the manufacturer, where 3 g/kg ethanol = 3.4 mL/kg, and 5 g/kg = 5.7 mL/kg.

### 3.3. Collection of Samples

After the last day of treatment; rats were fasted overnight, then anesthetized by thiopental sodium (50 mg/kg). Blood samples were collected from the retro-orbital plexus and centrifuged at 3000 rpm for 15 min for serum separation. Sera were stored at −20 °C for biochemical measurements.

Anesthetized rats were euthanized by cervical dislocation. Liver and kidney were isolated from each rat and washed with ice cold saline. The liver and kidney tissues of each rat were divided into two portions; the first was fixed in 10% neutral formalin for further histopathological processing, and the other portion was kept at −80 °C for further assessments.

### 3.4. Determination of Liver and Kidney Function Markers in Serum

The levels of the liver enzymes ALT, AST, and ALP were measured in the serum samples by colorimetric commercial kits (Ref. No. 264002, 260002 and 216001, respectively) (Spectrum Diagnostics, Cairo, Egypt). The serum levels of the kidney function parameters creatinine and BUN were also detected using colorimetric assay kits (Ref. No. 235001 and 318001, respectively) (Spectrum Diagnostics, Cairo, Egypt). Levels of iron in serum were determined by colorimetric kit (Cat. No. IR1510) (Bio-diagnostics, Giza, Egypt). Serum levels of ferritin were assayed by ELISA (Cat. No. ab157732) (Abcam, Cambridge, UK).

### 3.5. Assessment of Markers of Oxidative Stress and Inflammation in the Liver Tissue

Liver tissue was homogenized with ice cold saline. The homogenate of the liver was divided into three aliquots. The first aliquot was deproteinized with ice cold 12% trichloroacetic acid (TCA) followed by centrifugation at 4000 rpm for 15 min at 4 °C. GSH was detected in the resulting supernatant by a colorimetric kit (GR2511) (Bio-diagnostics, Egypt). The second aliquot was used to prepare a cytosolic fraction of the liver by centrifugation at 12,000 rpm for 15 min at 4 °C. The clear supernatant representing the cytosolic fraction was used for measurement of malondialdehyde (MDA), catalase, and superoxide dismutase (SOD) by colorimetric methods (Cat. No. MD2529, CA2517 and SD2521, respectively) (Bio-diagnostics, Giza, Egypt). The third aliquot was centrifuged at 4000 rpm for 15 min at 4 °C. Resulting supernatant was used to determine TNF-α and IL-6 levels in the liver tissue by ELISA (Cat. No. MBS2507393 and MBS175908, respectively) (MyBioSource, San Diego, CA, USA), according to the manufacturer’s protocol.

### 3.6. Quantitative Assessment of the Expression of Nuclear Factor Kappa B (NF-κB) and Hepcidin by Quantitative Real-Time PCR

Total RNA was extracted from portions of the frozen liver and kidney tissues by the RNeasy mini kit (Cat. No. 217004) (Qiagen, Hilden, Germany). Isolated RNA was converted into cDNA by the High-Capacity cDNA Reverse Transcription Kit (Cat. No. 4368814) (Applied Biosystems, Waltham, MA, USA). Real-time PCR was conducted to assess the hepcidin expression in liver tissue and the NF-κB expression in both liver and kidney tissues. The 20 μL reaction mixture consisted of 2 μL of the cDNA template (≈50 ng), 1 μL of each of the forward and the reverse primers (200 nM), 6 μL of nuclease free water, and 10 μL of the SYBR Green PCR Master Mix (Cat. No. 4309155) (Applied Biosystems, Waltham, MA, USA). The primers used for determination of the expression of NF-κB were: 5′-AATTGCCCCGGCAT-3′ (forward) and 5′-TCCCGTAACCGCGTA-3′ (reverse) [68]. The primers used for determination of hepcidin expression were: 5′-TGTCTCCTGCTTCTCCTCCT-3′ (forward) and 5′-CTCTGTAGTCTGTCTCATCTGTTG-3′ (reverse) [69]. Glyceraldehyde 3-phosphate dehydrogenase (GAPDH) was used as an endogenous control using the following primers: 5′-ATGACTCTACCCACGGCAAG-3′ (forward) and 5′-GATCTCGCTCCTGGAAGATG-3′ (reverse) [70]. The real time PCR reaction was conducted in a StepOnePlus thermal cycler (Applied Biosystems, Waltham, MA, USA). Cycling conditions involved initial denaturation at 95 °C for 10 min followed by 40 cycles of: denaturation at 95 °C for 30 s, annealing at 55 °C (NF-κB)/60 °C (hepcidin)/56 °C (GAPDH) for 30 s, and extension at 72 °C for 30 s. The PCR cycle was ended by final extension at 72 °C for 5 min. The cycle threshold (Ct) was recorded for each sample; ΔCt and the fold change were calculated.

### 3.7. Determination of Markers of Renal Inflammation and Injury in the Kidney Tissue

The stored kidney tissues were homogenized with ice cold saline. The homogenate was centrifuged at 4000 rpm for 15 min. Resulting supernatant was used to determine the levels of: TNF-α, KIM-1, vanin-1 and Cyt C by ELISA (MBS2507393, MBS355395, MBS9139552 and MBS727663, respectively) (MyBioSource, San Diego, CA, USA), according to the manufacturer’s instructions.

### 3.8. Histopathological Examination of the Liver and the Kidney

Sections from the liver and kidney of rats from different groups were fixed in 10% formalin saline for 24 h. Sections were then washed with tap water followed by serial dilutions of alcohol, cleared in xylene and embedded in paraffin to form blocks. A total of 56 blocks were prepared and sectioned at a thickness of 4 μm by sledge microtome. The tissue sections were collected on glass slides, deparaffinized with xylene, and stained by hematoxylin and eosin (H&E) for examination by an electric light microscope (Olympus, Shinjuku City, Tokyo, Japan). Histopathological scoring of hepatic inflammation and fibrosis were performed by an expert pathologist using METAVIR scoring [71]. In renal tissue, tubular injury score was determined based on the percentage of tubules with evident cell necrosis, loss of brush border, cast formation, and tubule dilatation, as follows: 0 = none, 1 = ≤10%, 2 = 11–25%, 3 = 26–45%, 4 = 46–75%, and 5 = ≥76% [72].

### 3.9. Statistical Analysis

Data were expressed as mean ± standard deviation (SD). Statistical analysis was performed by GraphPad Prism software (version 6.0). Values of the determined parameters in different groups were compared by one-way analysis of variance (ANOVA) followed by Bonferroni’s post-hoc test for multiple comparisons. All the reported *p* values were two-tailed and the differences were considered significant at *p* < 0.05.

## 4. Conclusions

To conclude, the findings of the current study suggest a protective role of LPS-RS, a potent TLR4 antagonist, against the hepatotoxicity and renal toxicity induced by both moderate and high doses of ethanol in experimental rats. To the best of our knowledge, this is the first study to report a protective effect of LPS-RS against ethanol induced toxicity in both liver and kidney. The effects of LPS-RS were executed through enhancement of the anti-oxidant capacity and attenuation of TLR4 downstream induction of inflammatory mediators. Additionally, LPS-RS helped to maintain iron homeostasis in the liver.

## Figures and Tables

**Table 1:** The effect of LPS-RS (800 μg/kg) on serum levels of liver enzymes and kidney function markers in the experimental rats.

	ALT (U\L)	AST (U\L)	ALP (U\L)	BUN (mg\dL)	Creatinine (mg\dL)
Intact control	42.0 ± 4.7	51.8 ± 5.3	53.0 ± 6.9	19.6 ± 2.1	0.9 ± 0.2
LPS-RS (800 μg/kg)	45.5 ± 5.2	57.7 ± 6.9	55.9 ± 6.6	21.0 ± 2.5	1.1 ± 0.2
Ethanol (3 g/kg)	105.0 ± 15.5 ^ab^	225.6 ± 22.2 ^ab^	185.0 ± 19.1 ^ab^	27.9 ± 1.9 ^ab^	1.6 ± 0.3 ^ab^
LPS-RS + ethanol (3 g/kg)	55.0 ± 6.0 ^abc^	92.3 ± 9.5 ^abc^	56.6 ± 6.0 ^c^	26.6 ± 2.4 ^ab^	1.1 ± 0.3 ^c^
Ethanol (5 g/kg)	162.4 ± 17.1 ^abcd^	265 ± 27.7 ^abcd^	248.7 ± 25.8 ^abcd^	28.3 ± 2.1 ^ab^	1.9 ± 0.2 ^abd^
LPS-RS + ethanol (5 g/kg)	99.0 ± 11.2 ^abde^	204.4 ± 21.9 ^abde^	115.6 ± 12.6 ^abcde^	26.7 ± 1.3 ^ab^	1.4 ± 0.3 ^abe^

Data are expressed as mean ± SD and analyzed using one-way ANOVA followed by Bonferroni’s post hoe test (*n* = 8–10). ALT = alanine amino-transferase; AST = aspartate amino-transferase; ALP = alkaline phosphatase, BUN = blood urea nitrogen; Differences were considered significantly different at *p* < 0.05. ^a^ vs. intact control; ^b^ vs. LPS-RS; ^c^ vs. ethanol (3 g/kg); ^d^ vs. LPS-RS + ethanol (3 g/kg); ^e^ vs. ethanol (5 g/kg).

## Data Availability

The datasets used and analyzed are available from the corresponding author on reasonable request.
